# Carboplatin/paclitaxel, E7-vaccination and intravaginal CpG as tri-therapy towards efficient regression of genital HPV16 tumors

**DOI:** 10.1186/s40425-019-0593-1

**Published:** 2019-05-06

**Authors:** Sonia Domingos-Pereira, Gabriele Galliverti, Douglas Hanahan, Denise Nardelli-Haefliger

**Affiliations:** 1 Department of Urology, Centre Hospitalier Universitaire Vaudois, Bugnon 48, 1011 Lausanne, Switzerland; 2 Swiss Institute for Experimental Cancer Research, School of Life Sciences, EPFL, 1015 Lausanne, Switzerland

**Keywords:** HPV genital cancer, Carboplatin/paclitaxel, E7 long peptide and nanoparticle vaccination, Intravaginal CpG immunostimulation

## Abstract

High-risk human papillomavirus (HPV) are responsible for genital and oral cancers associated with the expression of the E6/E7 HPV oncogenes. Therapeutic vaccines targeting those oncogenes can only partially control tumor progression, highlighting the necessity to investigate different treatment strategies. Using the genital orthotopic HPV16 TC-1 model, herein we sequentially investigated in progressively more stringent settings the effects of systemic administration of carboplatin/paclitaxel (C + P) chemotherapy combined with HPV16-E7 synthetic long peptide (E7LP) vaccination, followed by intravaginal immunostimulation with the synthetic toll-like-receptor-9 agonist CpG. Our data show that systemic delivery of C + P prior to E7LP vaccination significantly increased mice survival. This survival benefit was associated with both reduced genital tumor growth at the time of vaccination, and a decreased infiltration of Ly6G myeloid cells and tumor-associated macrophages. Adding intravaginal CpG, which results in increased E7-specific CD8 T cells locally, to E7LP vaccination and the chemotherapy formed a tri-therapy, which significantly increased mice survival as compared to any of the dual treatments. When the tri-therapy was further refined by using a recently optimized nanoparticle-conjugated E7LP vaccine, even larger end-stage genital-TC-1 tumors responded, with 90% of mice showing a survival benefit as compared to 30% of mice with the tri-therapy involving the traditional E7LP ‘liquid’ vaccine. C + P is commonly used to treat cervical cancer patients and its combination with E7/E6 vaccination is currently being tested in a phase I/II trial (NCT02128126). Our data suggests that new vaccine formulations combined with local immunostimulation and standard-of-care chemotherapy have promise to further benefit patients with HPV-associated cancer.

**Electronic supplementary material:**

The online version of this article (10.1186/s40425-019-0593-1) contains supplementary material, which is available to authorized users.

## Introduction

Human papilloma virus (HPV)-associated cancers i.e. all cervical cancers and part of vaginal, vulvar, anal and oral cancers, result from infection of the basal cells of the stratified epithelium by high-risk HPV types [1]. Because expression of E6 and E7 HPV oncogenes in cervical epithelial cells is required for the maintenance of the cancerous phenotype, they represent attractive target antigens for therapy [1]. Therapeutic HPV vaccines designed to induce specific cytotoxic T-cell responses against tumor cells have shown impressive results in subcutaneous (s.c.) animal models. Initial application in humans has shown modest clinical effectiveness in the last 20 years and no candidate vaccine has yet demonstrated sufficient efficacy in inducing regression of HPV-induced cancer to warrant commercialization. However, therapeutic vaccines have been considerably improved with several ongoing clinical trials [2], including the development of synthetic long peptides (LP) vaccines [3]. Along this line we recently showed that conjugation of HPV16-E7LP to ultra-small polymeric nanoparticles (NP) enhanced the antitumor efficacy of therapeutic vaccination in different mouse models of HPV^+^ cancers [4]. However, combination with different treatment modalities are clearly necessary towards future successes [3]. Of particular interest is the combination of carboplatin/paclitaxel (C + P) chemotherapy, commonly used to treat cervical cancer patients, with HPV-E7/E6LP vaccination which showed promising results in the subcutaneous (s.c.) HPV-expressing TC-1 model [5] and currently being tested in a phase I/II trial (NCT02128126). Taking advantage of the accessibility of the genital mucosa, here we further investigated how local immunostimulation may provide additional benefit to E7-vaccination and C + P chemotherapy in mice bearing genital TC-1 tumors, as a model that may represent more reliably the clinical situation, at least in regards to the local cervico-vaginal tumor microenvironment [6]. We previously showed in this model, but also in bladder cancer models, that in systemically vaccinated mice, intravaginal or intravesical administration of different synthetic or bacterial immunostimulants both local vaccine-specific CD8 T-cell responses and tumor regression were enhanced [7–9]. More importantly, we provided proof of principle efficacy of such strategy in non-muscle invasive bladder cancer patients where intravesical Bacille Calmette Guerin immunotherapy was combined to systemic immunization with a cancer vaccine [10]. Using the genital orthotopic TC-1 model, we therefore here sequentially investigated in progressively more stringent settings the effects of systemic administration of C + P chemotherapy and E7LP or NP-E7LP vaccination, followed by intravaginal immunostimulation with the synthetic toll-like receptor-9 agonist CpG.

## Results and discussion

### Carboplatin-paclitaxel (C + P) chemotherapy decreased genital tumor size and a potential immunosuppressive tumor microenvironment for a better vaccine efficacy

**Fig. 1:**
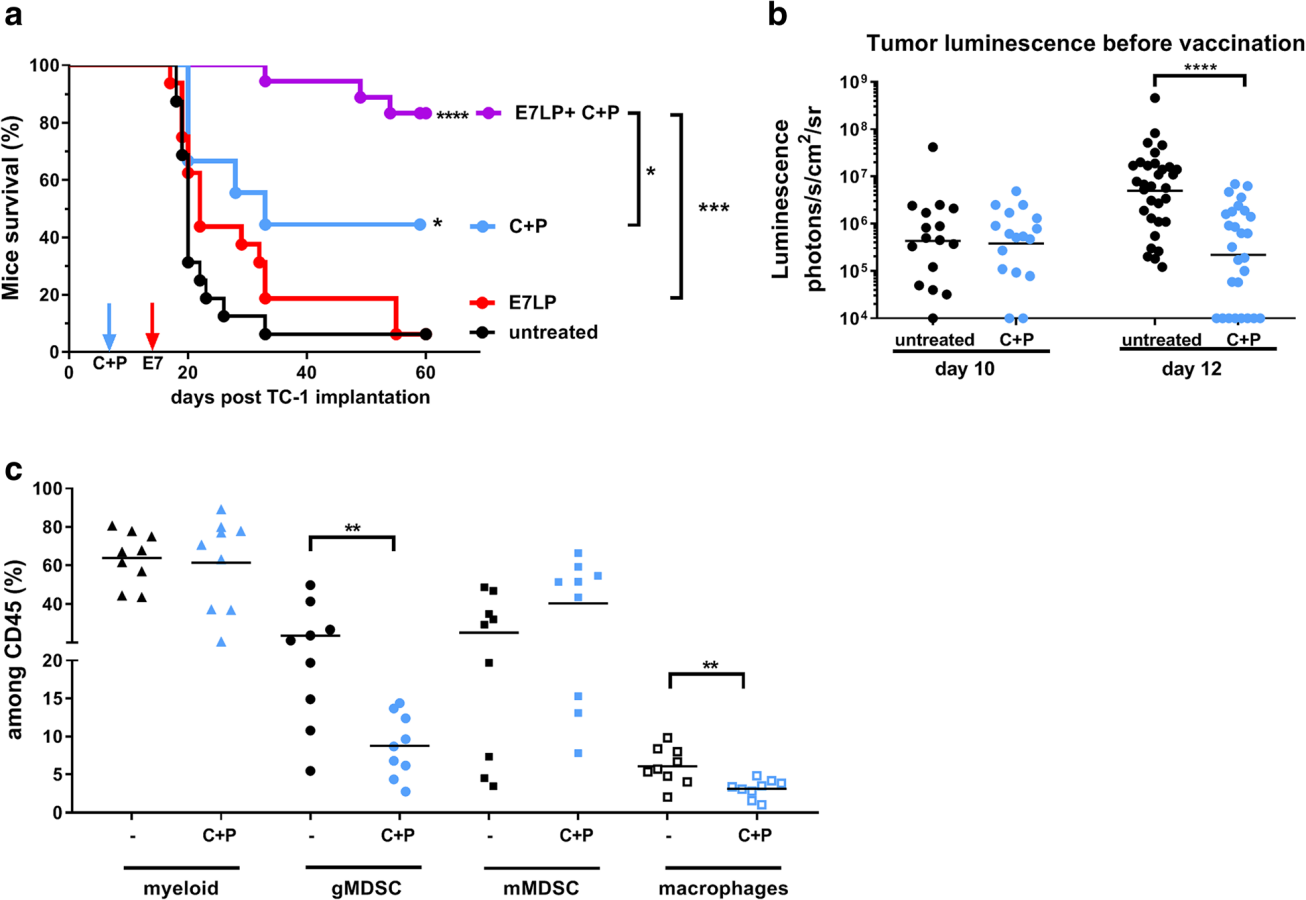
Effect of C + P on tumor size and E7LP vaccination. Groups of female mice bearing TC-1 genital tumors received i.p. 1 dose of Carboplatin (40 mg/kg) at day 8 and 2 doses of paclitaxel (20 mg/kg at days 8/9 and/or s.c. vaccination with E7 vaccine (E7LP) at day 13. Mice survival (**a**) and tumor luminescence before vaccination (**b**) are shown. C: Flow-cytometry analysis of TC-1 genital tumors untreated or treated with C + P as indicated on the graphs is shown at day 13. Individual and mean % among CD45^+^ of myeloid cells (CD45^+^CD11b^+^), gMDSC (CD45^+^CD11b^+^Ly6G^+^), mMDSC (CD45^+^CD11b^+^Ly6C^+^) and macrophages (CD45^+^CD11b^+^Ly6G^−^Ly6C^−^F4/80^+^) are shown. Significant differences are shown following a log rank test (**a**) or a Student t-test (**b**-**c**): * = *p* < 0.05, ** = *p* < 0.01, *** = *p* < 0.001, **** = *p* < 0.0001

We first examined the effect of C + P chemotherapy on E7LP vaccination in the genital TC-1 model. Groups of female mice bearing TC-1-luc genital tumors received intraperitoneally (i.p.) one dose of Carboplatine (40 mg/kg) at day 8 and two doses of Paclitaxel (20 mg/kg) at days 8 and 9 (C + P chemotherapy) and/or s.c. vaccination with E7LP at day 13. Combination of C + P and E7LP resulted in a significantly enhanced survival as compared to E7LP vaccination alone (Fig. 1a). Interestingly, 4 days after CP treatment, at day 12, genital tumor size, as evaluated by bioluminescence imaging, was significantly smaller than in untreated mice (Fig. 1b). This result suggests that C + P can directly affect tumor growth in agreement with taxanes (Paclitaxel) and platinium compounds (Carboplatin) being able to inhibit mitosis and DNA replication, respectively [11, 12]. In addition, C + P can also enhance anti-tumor mechanisms [11, 12] and experiment on s.c. TC-1 tumor, showed that C + P was rather decreasing circulating and tumor-infiltrating CD11b^+^ GR1^high+^/or Ly6G^+^ myeloid cells [5]. Thus, immune cell infiltration in genital TC-1 tumors (day 13) was evaluated by flow cytometry (see gating strategy and representative cytometry plots in Additional file 1) in mice bearing TC-1 genital tumors that had received either C + P treatment (day 8/9) or left untreated. C + P significantly reduced the infiltration of Ly6G^+^ myeloid cells (a phenotype of potential granulocytic myeloid derived suppressor cells, hereafter referred as gMDSC), similarly to data obtained in s.c.TC-1 tumors [5]; but also of macrophages, while Ly6C^+^ myeloid cells (a phenotype of potential monocytic MDSC, hereafter referred as mMDSC) were not affected (Fig. 1c). Similarly, circulating gMDSC in these mice were also decreased upon C + P (see Additional file 2). These data suggests that a dual effect of C + P on tumor-growth and on the tumor microenvironment may participate in vaccine efficacy.

### Intravaginal immunostimulation with CpG after vaccination resulted in a more effective tri-therapy

**Fig. 2:**
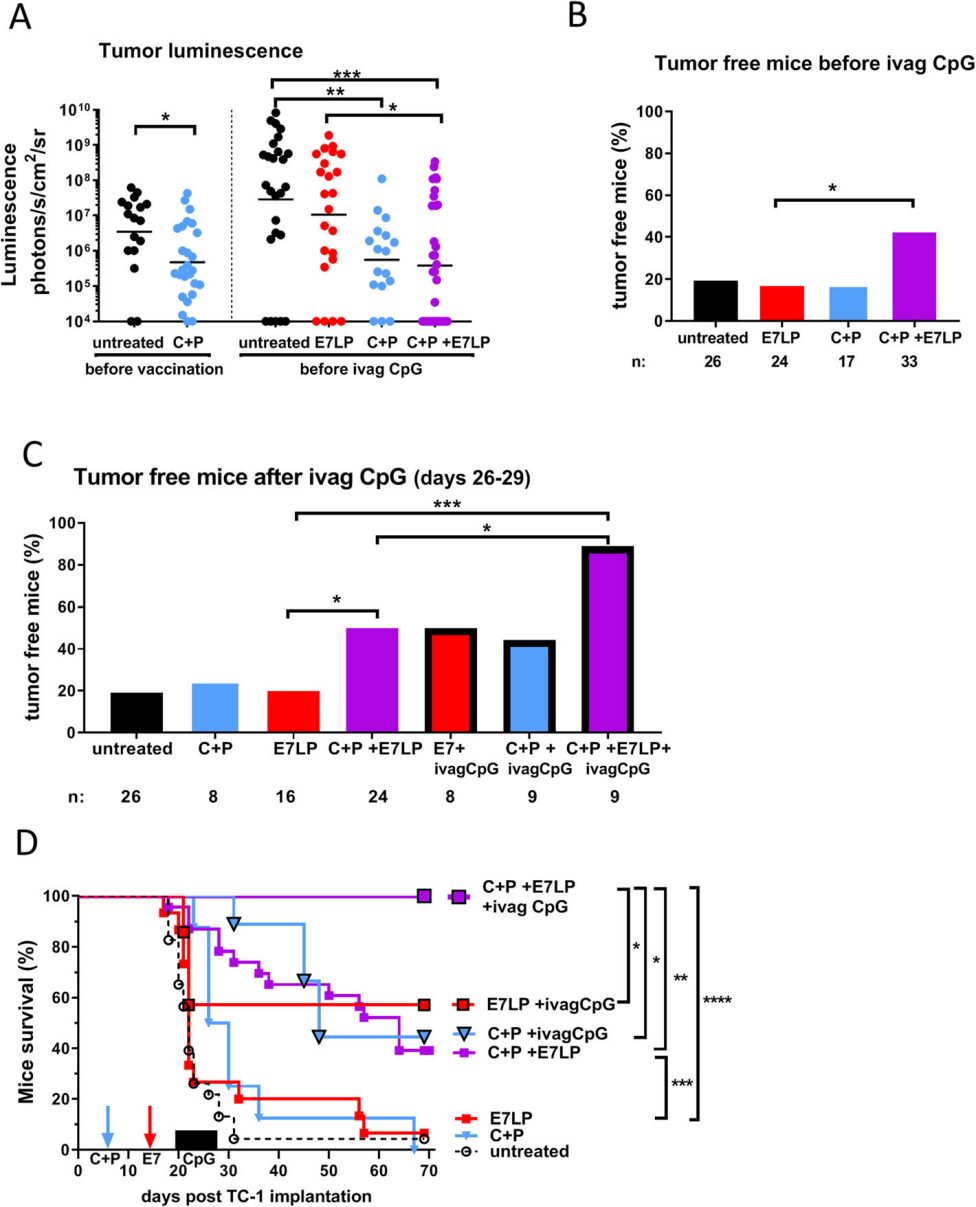
Intravaginal (ivag) immunostimulation with CpG after C + P and E7LP vaccination resulted in an effective tri-therapy. Groups of female mice bearing TC-1 genital tumors received different treatments as indicated on the graphs. C + P (days 8/9) and/or vaccination with E7LP (day 15) and /or ivag administration of CpG (at days 20, 23 and 26). Tumor luminescence before vaccination or before ivag CpG (**a**), percentages of tumor free mice before ivag CpG (**b**) and after ivag CpG (days 26–29) (**c**) and mice survival (**d**) are shown. Total number of mice/group (n) are indicated (**b**-**c**). Significant differences following one-way Anova +Tukey’s post-test (**a**), contingency Chi-square test (**b**-**c**) or log rank test (**d**) are shown: * = *p* < 0.05, ** = *p* < 0.01, *** = *p* < 0.001, **** = *p* < 0.0001

Based on the schedule of our previous experiments with intravaginal (ivag) CpG immunostimulation [7], we next tested administration of ivag CpG (three 100 μg doses administered 5, 8 and 11 days after E7LP vaccination performed at day 15). The effect on tumor growth and mice survival was examined in groups of mice that received a single treatment (E7LP or C + P), bi-therapies (C + P + E7LP, C + P + ivag CpG or E7LP + ivag CpG), a tri-therapy (C + P + E7LP + ivag CpG) or left untreated. Comparison of tumor size before ivag CpG instillation (at day 20) confirmed the significant smaller tumor size in the mice that had received C + P (Fig. 2a). Interestingly, C + P + E7LP bi-therapy not only significantly decreased tumor size (Fig. 2a), but also increased the percentage of tumor-free mice, as compared to mice vaccinated with E7LP alone (Fig. 2b). When compared at days 26–29 (i.e. after ivag CpG for the concerned mice, Fig. 2c), 90% of the mice that had received the tri-therapy were tumor-free, as compared to ca. 50% for the mice that had received bi-therapies and ca. 20% for C + P only, E7LP only or untreated mice. The significantly higher efficacy of the tri-therapy as compared to any of the dual treatments was further confirmed for mice survival (Fig. 2d). Note that in this more stringent setting, where vaccination was performed at day 15 (as compared to day 13 in Fig. 1), the efficacy of C + P + E7LP bi-therapy only resulted in 40% mice survival at the defined endpoint (as compared to 90% survival at this time point when mice with smaller tumors were vaccinated, Fig. 1), thus demonstrating the necessity and the additional benefit of ivag CpG. Notably, it is the prolonged survival provided by C + P that allowed the delay in vaccination thereby providing a window of opportunity for performing ivag CpG treatment. We previously reported that ivag CpG after vaccination increased the number of vaccine-specific CD8 T-cells in the genital mucosa [7] and this was further confirmed in the present setting, with or without C + P prior to E7LP vaccination (see Additional file 3). However, the low number of E7-specific IFN-γ secreting cells measured in the genital mucosa two weeks after vaccination also confirmed the relatively low immunogenicity of the E7LP “liquid” formulation of this vaccine [4].

### Inclusion of a nanoparticle (NP)-conjugated E7 vaccine increased the tri-therapy efficacy

**Fig. 3:**
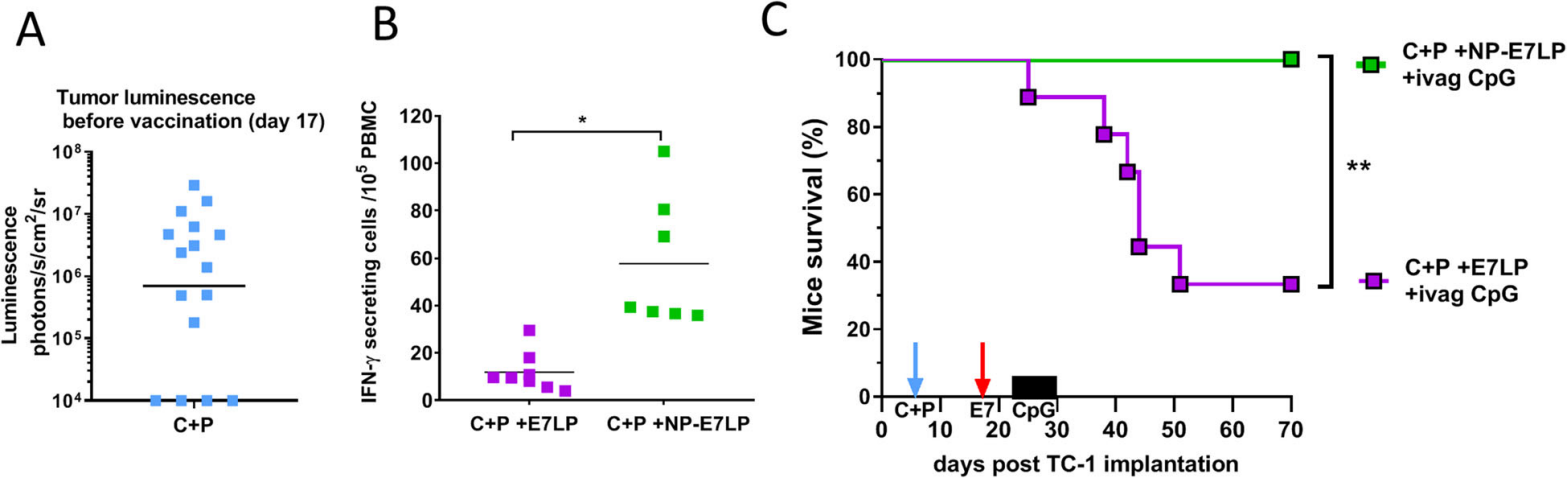
Including the nanoparticle vaccine (NP-E7LP) in the tri-therapy can efficiently regress very large TC-1 genital tumors. Groups of female mice bearing TC-1 genital tumors received C + P (days 8/9) and vaccination with E7LP or with NP-E7LP (at day 18) followed by ivag administration of CpG at days 23, 26 and 29. Tumor luminescence before vaccination (**a**), E7-specific T cells responses in PBMC one week after vaccination (**b**) and mice survival (**c**) are shown. Significant differences following a student t-test (**b**) or a log rank test (**c**) are shown: * = *p* < 0.05, ** = *p* < 0.01

Finally, we evaluated a newly developed NP-conjugated E7LP vaccine [4] to be included in our tri-therapy (C + P + NP-E7LP + ivag CpG) for treating even larger genital TC-1 tumors (day 18, Fig. 3a). Analysis by IFN-γ ELISPOT of peripheral blood mononuclear cells (PBMC) of the genital-tumor bearing mice 6 days after vaccination (24 h after the 1st ivag CpG) revealed a significantly higher E7-specific CD8 T-cell response induced by NP-E7LP as compared to E7LP (Fig. 3b). When the tri-therapy included NP-E7LP, survival benefit at a defined endpoint of 60 days was markedly increased (100%) in comparison to the tri-therapy including the conventional E7LP formulation (30%, Fig. 3 c) demonstrating the benefit of this solid phase vaccine in the context of the refined tri-therapy involving C + P chemotherapy and ivag CpG immune-stimulant.

## Conclusion

The introduction and increasingly widespread application of prophylactic capsid HPV vaccines will in future decades certainly reduce the incidences of both cervical and oral cancer, however these vaccines provide no benefit for individuals already infected with HPV. Recognizing therefore the continuing need for therapies to more effectively treat HPV-induced cervical and oral cancers, here we report on the development and evaluation of a regimen that combines chemotherapies currently in use in cervical cancer patients, along with an optimized therapeutic E7LP vaccine and a localized immune-stimulant treatment. In the clinics, C + P chemotherapy is usually given every 3 weeks (for 6 cycles), a period that would allow inclusion of E7-vaccination + ivag immunostimulations scheduled as in the mouse pre-clinical setting. The fact that vaccination and local immunostimulations may mainly lead to local discomfort, which should not exacerbate the side effects already induced by C + P chemotherapy, should favor the implementation of such tri-therapies. In this study, we used a synthetic TLR-9 agonist, CpG, but topical immunostimulation with a TLR-7 agonist (Imiquimod/Aldara®) has been used for some time in patients with benign genital HPV-lesions and has shown benefit in conjunction with E7/E6 vaccination in patients with vulvar intraepithelial neoplasia [13]. An interesting alternative to synthetic TLR agonists for local immunostimulation may be the use of bacterial vaccine, as we reported with intravesical BCG in bladder cancer patients [10]. Another bacterial vaccine, the attenuated Salmonella Ty21a typhoid-fever vaccine (Vivotif®), may be of even greater interest because of its higher safety [14] and the larger infiltration of vaccine-specific CD8 T-cells, as we previously reported in the genital mucosa [8] and in the bladder [9] after local instillation in mice. The high safety profile of the oral Ty21a vaccine was shown worldwide in more than 200 million vaccinees over the last 30 years [14] and we are now assessing safety of intravesical instillations in bladder cancer patients in our hospital (NCT 03421236: IVES Ty21a), towards topical applications on other mucosal sites. Finally, it is evident from this and other studies that solid phase nanoparticle and nanogels that afford more persistent immune-stimulation with peptide antigens than conventional liquid formulations hold promise, and warrant further evaluation as potential components of the differentially efficacious tri-therapy illustrated herein.

## Material and methods

### Mice treatments

Seven to ten-week-old female C57Bl/6 wild type mice (Charles River and Envigo) were used and all experiments were performed in accordance with Swiss law and with approval of the Cantonal Veterinary Office of Canton de Vaud, Switzerland. The TC-1 cell line (C57BL/6 primary lung epithelial cells transduced with retroviral vectors expressing HPV16 E6/E7 and activated c-Ha-ras) kindly provided by Prof. T.-C. Wu (Johns Hopkins Medical Institutions, Baltimore, USA) was transduced with a luciferase-expressing lentiviral vector to generate TC-1-luc cells used to establish genital tumors [15]. For this purpose, anesthetized diestrus synchronized-mice (E/DP: s.c. injection with 0.1 μg β-estradiol, Sigma Merck KGaA, Darmastadt, Germany, and 24 h later with 2 mg DepoProvera, Pfizer AG, Zurich, Switzerland: one and two weeks before TC-1 luc challenge) were ivag pre-treated with 4% nonoxynol-9 (N9, Igepal, Sigma Merck KGaA) for 6 h, washed with PBS and ivag challenged with 50′000 TC-1-luc cells (day 1). Genital tumor growth was monitored by bioluminescence 15 min after an i.p. injection of D-luciferin (Promega, Wisconsin, USA) 150 μg/g of body weight) in a Xenogen imaging system (IVIS lumina, Xenogen/Caliper Life Science, kindly provided by Cellular Imaging Facility, UNIL, Lausanne). C + P chemotherapy consisted in i.p. administration of one dose of Carboplatine (40 mg/kg, Actavis, New-jersey, USA) at day 8 and two doses of Paclitaxel (20 mg/kg, Labatec Pharma, Meyrin, Switzerland) at days 8 and 9.

A single s.c. vaccination was performed at days 13, 15 or 18 after ivag TC-1 luc challenge with E7LP (15 μg of synthetic E7_43–77_ peptide, chemically synthesized by the Protein and Peptide Chemistry Facility of the Institute of Biochemistry, UNIL, Switzerland, and 40 μg of CpG: CpG-B 1826 oligonucleotide 5′-TCCATGAGCTTCCTGACGTT-3′ as phosphorothioated DNA bases, purchased from Microsynth) or at day 18 with NP-E7LP, the NP conjugated vaccine prepared as detailed in [4]. Briefly, NPs were synthesized, functionalized, and characterized and then incubated for 12 h in endotoxin-free water and guanidine hydrochloride with E7_43–77_ dissolved in DMSO. NP-E7 was purified by size-exclusion chromatography using CL-6B matrix (Sigma Merck KGaA), eluted, and stored in PBS at room temperature, 40 μg of CpG were added before vaccination.

For ivag immunostimulation CpG (100 μg/dose, 3 doses, 3 days apart, starting 5 days after vaccination) were instilled (10 μl volume) using a micropipette tip in the vagina of deeply anesthetized mice which were kept in diestrus stage after vaccination by additional E/DP treatments at days 10/11 and 16/17.

### Organ preparation

Mice were sacrificed by CO2 inhalation to collect the cervico-vaginal tumor tissues. Single-cell suspensions were obtained by mincing in DL-dithiothreitol (Sigma Merck KGaA) and digesting with 1 mg/mL collagenase/dispase (Roche, Basel, Switzerland) and 0.1 mg/ml DNAse I (Sigma Merck KGaA) with 20% Fetal Calf Serum (Gibco, MA, USA). Whole blood was collected in tubes containing heparin-Na 25,000 I.E. (Braun, Sempach, Switzerland) from tail vein. Red blood cells were lysed using ammonium-chloride-potassium. PBMC were prepared from tail-blood [16]. The recovered cells were used for immunostaining and/or for IFN-γ ELISPOT assay.

### Immunostaining and flow cytometry analysis

The monoclonal anti-mouse antibodies used were: Anti-CD3-PE (17A2), Anti-CD3-PerCP/Cy5.5 (17A2), Anti-Ly6G-PE/Cy7 (1A8), Anti-Ly6G-APC/Cy7 (1A8), Anti-CD11b-APC (M1/70), Anti-Ly6C-AF700 (HK1.4), Anti-CD8-APC/Cy7 (53–6.7), Anti-F4/80-APC-Cy7 (BM8) (Biolegend, CA, USA); Anti-CD4-FITC (GK1.5), Anti-CD45-FITC (30-F115), Anti-CD45-PerCP/Cy5.5 (30-F11), Anti-CD11b-eF450 (M1/70) (eBioscience, Thermo Fisher Scientific, MA, USA). The following Isotype control was used: rat IgG2a, κ isotype control-APC-Cy7 (RTK2758, Biolegend). Dead cells were excluded by a live/dead fixable aqua dead cell stain kit (Invitrogen Thermo Fisher Scientific, MA, USA). Cell acquisition and analysis were performed using Gallios Flow Cytometer (Beckman Coulter, Nyon, Switzerland) and FlowJo software (Tree Star, Ashland, OR), respectively.

### IFN-γ ELISPOT assay

IFN-γ ELISPOT assay were performed as described in detail in [16]. Briefly, 30′000 bone marrow derived dendritic cells/well were loaded with 1 μg/ml of E7_49–57_ peptide or medium alone (control wells) for 1 h before adding 200′000 CV cells/well. After 16 to 24 h. E7-specific responses were defined for each individual mouse as the number of IFN-γ spots/10^5^ cells in the E7-stimulated wells minus the number of IFN-γ spots/10^5^ cells in the control wells.

### Statistics

Statistical analyses were performed using Prism 7.00 for Windows (GraphPad software). Single comparisons were performed using t-test. Multiple comparisons were performed using one-way Anova and Dunnets’s post- test or log-rank test as indicated in the figure legends.

## Additional files

Additional file 1: Effect of C+P on myeloid cell infiltration. (PDF 308 kb)

Additional file 2: Effect of C+P on circulating myeloid cell. (PDF 290 kb)

Additional file 3: Ivag CpG after E7LP or C+P +E7LP vaccination increased E7-specific IFN-γ secreting cells in Cervix-Vagina (CV). (PDF 203 kb)

## Abbreviations

CP: carboplatin/paclitaxel
gMDSC: granulocytic myeloid derived suppressor cells
HPV: Human papilloma virus
ivag: intravaginal
LP: synthetic long peptide
mMDSC: monocytic myeloid derived suppressor cells
NP: nanoparticle
s.c: subcutaneous
