# Supplementary material for: Carboplatin/paclitaxel, E7-vaccination and intravaginal CpG as tri-therapy towards efficient regression of genital HPV16 tumors
Source: J Immunother Cancer. 2019 May 6;7:122. doi: 10.1186/s40425-019-0593-1 (PMC6503370; doi:10.1186/s40425-019-0593-1)
Supplement: Supplementary file 1 — Effect of C+P on myeloid cell infiltration. (PDF 308 kb) [file 40425_2019_593_MOESM1_ESM.pdf]

Additional File 1

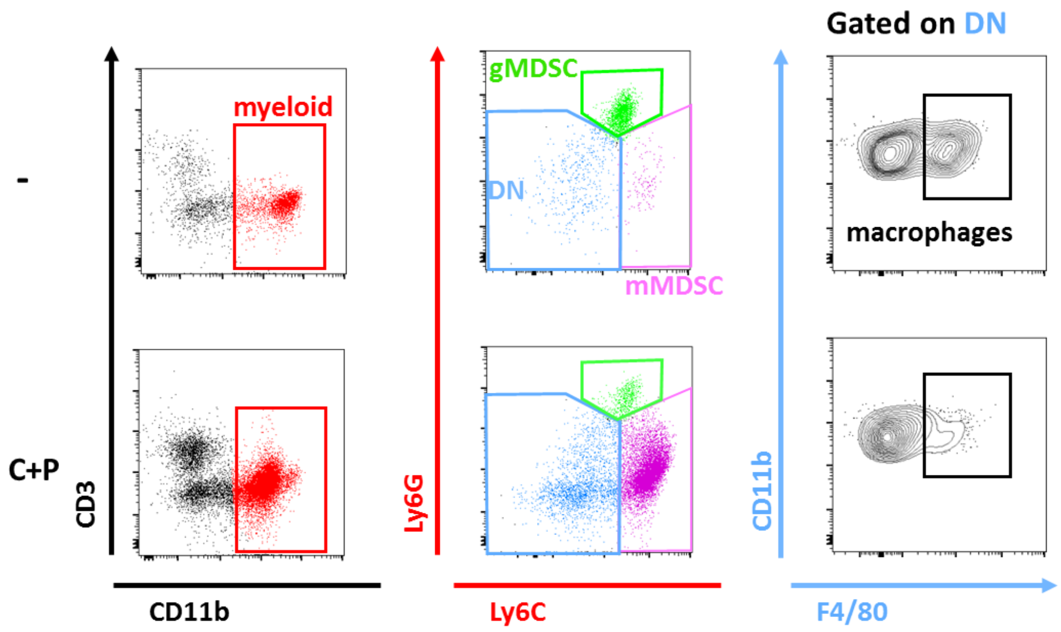

Additional File 1. Effect of C+P on myeloid cell infiltration. Gating strategy and representative flow cytometry plots showing myeloid ($CD11b^{+}$), gMDSC ($CD11b^{+}Ly6G^{+}$), mMDSC ($CD11b^{+}Ly6C^{+}$), double negative (DN : $CD11b^{+}Ly6G^{neg}Ly6C^{neg}$) and macrophage ($CD11b^{+}Ly6G^{neg}Ly6C^{neg}F4/80^{+}$) cell infiltration of TC-1 genital tumors at day 13 after C+P treatment or left untreated.
