# Supplementary material for: Carboplatin/paclitaxel, E7-vaccination and intravaginal CpG as tri-therapy towards efficient regression of genital HPV16 tumors
Source: J Immunother Cancer. 2019 May 6;7:122. doi: 10.1186/s40425-019-0593-1 (PMC6503370; doi:10.1186/s40425-019-0593-1)
Supplement: Supplementary file 2 — Effect of C+P on circulating myeloid cell. (PDF 290 kb) [file 40425_2019_593_MOESM2_ESM.pdf]

Additional File 2

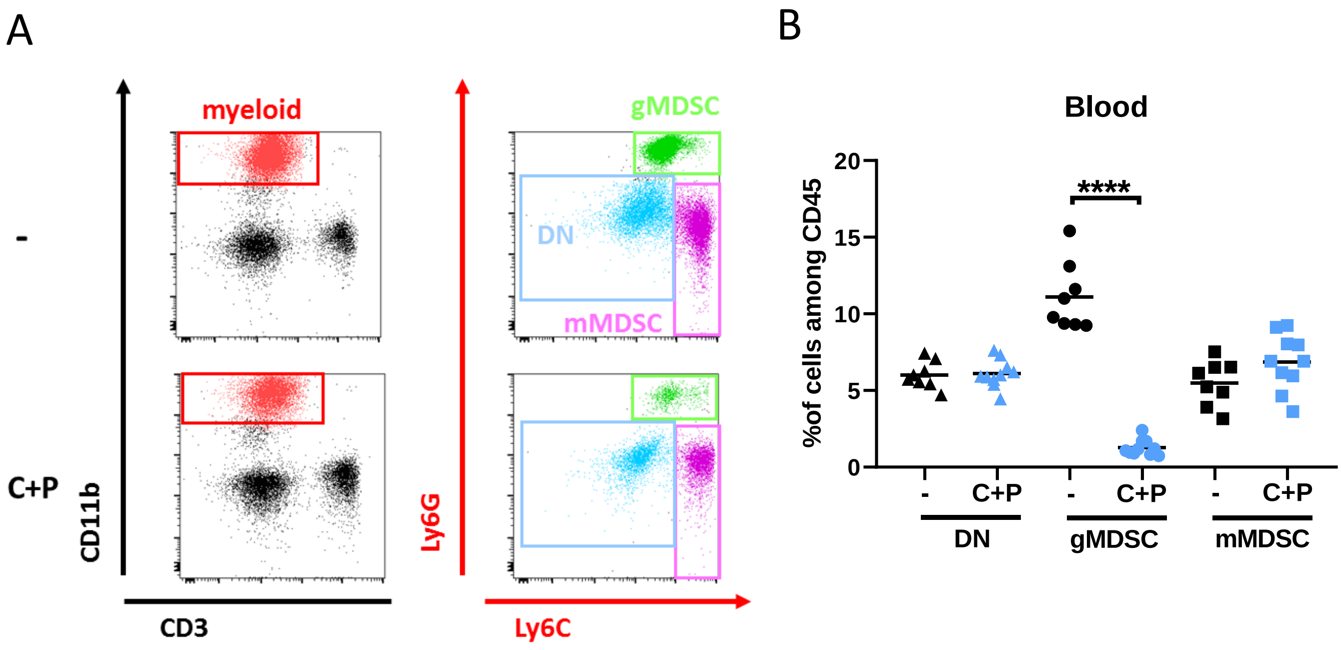

Additional File 2. Effect of C+P on circulating myeloid cells. A: Gating strategy and representative flow cytometry plots showing myeloid (CD11b^{+}), gMDSC (CD11b^{+}Ly6G^{+}), mMDSC (CD11b^{+}Ly6C^{+}) and double negative (DN, CD11b^{+}Ly6G^{neg}Ly6C^{neg}) cells in whole blood of mice bearing day 13 TC-1 genital tumors after C+P treatment or left untreated. **B** mean and individual percentages of the indicated myeloid cell subtypes among CD45^{+} cells.
