# Supplementary material for: Carboplatin/paclitaxel, E7-vaccination and intravaginal CpG as tri-therapy towards efficient regression of genital HPV16 tumors
Source: J Immunother Cancer. 2019 May 6;7:122. doi: 10.1186/s40425-019-0593-1 (PMC6503370; doi:10.1186/s40425-019-0593-1)
Supplement: Supplementary file 3 — Ivag CpG after E7LP or C+P +E7LP vaccination increased E7-specific IFN-γ secreting cells in Cervix-Vagina (CV). (PDF 203 kb) [file 40425_2019_593_MOESM3_ESM.pdf]

Additional File 3

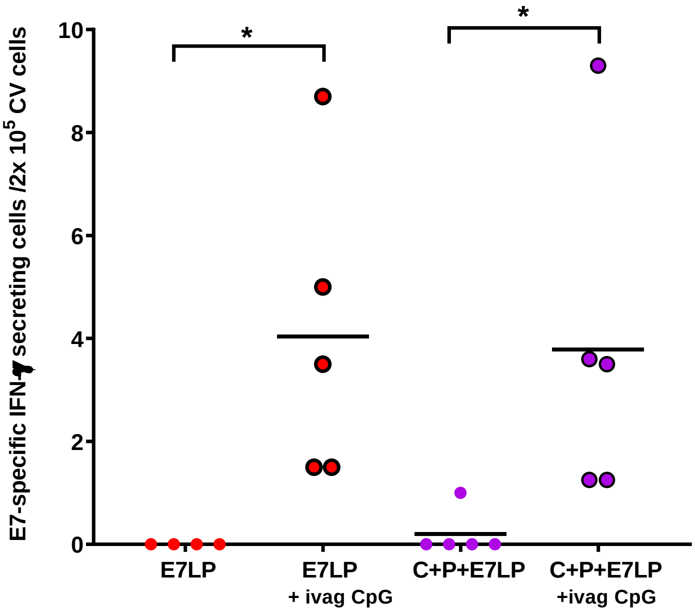

Additional File 3. Ivag CpG after E7LP or C+P +E7LP vaccination increased E7-specific IFN-γ secreting cells in Cervix-Vagina (CV). Groups of naive mice received the treatments indicated below the graph. C+P chemotherapy was administered 4/5 days before vaccination. 3 doses of ivag CpG were administered 5,8 and 12 days after vaccination. CV were analyzed by IFN-γ ELISPOT 15 days after vaccination. Mean and individual E7-specific INF-γ secreting T cells are shown. *=p<0,05 by Student t-test.
